# Nuclear Medicine During the COVID-19 Pandemic: The Show Must Go On

**DOI:** 10.3389/fmed.2022.896069

**Published:** 2022-05-12

**Authors:** Giorgio Treglia

**Affiliations:** ^1^Clinic of Nuclear Medicine, Imaging Institute of Southern Switzerland, Ente Ospedaliero Cantonale, Bellinzona, Switzerland; ^2^Department of Nuclear Medicine and Molecular Imaging, Lausanne University Hospital, Lausanne, Switzerland; ^3^Academic Education, Research and Innovation Area, General Directorate, Ente Ospedaliero Cantonale, Bellinzona, Switzerland; ^4^Faculty of Biology and Medicine, University of Lausanne, Lausanne, Switzerland; ^5^Faculty of Biomedical Sciences, Università della Svizzera Italiana, Lugano, Switzerland

**Keywords:** nuclear medicine (NM), COVID-19, hybrid imaging, imaging, research

The coronavirus disease 2019 (COVID-19) pandemic is an ongoing global emergency caused by severe acute respiratory syndrome coronavirus 2 (SARS-CoV-2). The novel virus was first identified from an outbreak in Wuhan (China) in December 2019, then the infection rapidly spread across the globe. The World Health Organization (WHO) declared COVID-19 a pandemic on 11 March 2020. As of 28 December 2021, the pandemic had caused more than 280 million cases and 5.4 million deaths (1).

The COVID-19 pandemic had an impact in several medical fields, including nuclear medicine. Nuclear medicine procedures are used for functional imaging of pathophysiological processes at cellular or molecular levels using specific diagnostic radiopharmaceuticals and for treatment of several diseases based on targeted delivery of therapeutic radiopharmaceuticals. COVID-19 has created several challenges related to organizational, clinical/imaging, and research settings in nuclear medicine.

Regarding organizational aspects, based on several international surveys and initiatives, it is currently well-known that the COVID-19 pandemic had a significant impact on the activities of nuclear medicine departments worldwide (2–11). For instance, the volume of nuclear medicine diagnostic and therapeutic procedures declined globally (for several reasons including reduced activity of some centers, postponements of non-urgent procedures, and patients' fear about COVID-19) with variable degrees of restoration and possible impact on patient management (2). COVID-19-adapted nuclear medicine guidelines for standard operating procedures (SOPs) were created, including infection protection measures for patients and nuclear medicine staff (2). Furthermore, the COVID-19 pandemic also negatively influenced the supply of radiopharmaceuticals to nuclear medicine departments; on the other hand, applications of communications technologies in nuclear medicine increased including telemedicine and education (2).

Regarding clinical/imaging aspects, hybrid imaging techniques may provide functional and morphological information for early diagnosis of infectious and inflammatory diseases (12), including COVID-19. Evidence-based data demonstrated that several nuclear medicine procedures and hybrid imaging techniques may incidentally detect COVID-19 lesions (e.g., interstitial pneumonia suspected for COVID-19) in patients who underwent these imaging techniques for oncological and non-oncological indications (13, 14). Notably, hybrid imaging methods are currently not used in the clinical practice to diagnose COVID-19 or for disease monitoring and they cannot substitute high-resolution computed tomography in this setting (15). However, incidental early diagnosis of COVID-19 through hybrid imaging techniques may be crucial for appropriate patient management (13, 14).

As of 28 December 2021, about 9 billion doses of COVID-19 vaccines have been administered worldwide (1). Incidental findings using hybrid imaging methods in patients who have received COVID-19 vaccinations, including hypermetabolic axillary lymph nodes ipsilateral to the COVID-19 vaccine injection site, have been widely described (16, 17). These imaging findings are frequent and they may cause diagnostic dilemmas, in particular, in oncological patients. Nuclear medicine physicians must be aware and recognize the significant frequency of hybrid imaging findings related to immune response to vaccine injection (16, 17).

Overall, COVID-19 and COVID-19 vaccination may cause a strong activation of the immune system leading to incidental hybrid imaging findings as local inflammation (frequent finding) or triggered autoimmune diseases (unusual finding) (18).

Regarding the research setting, the COVID-19 pandemic incentivized research activities in several medical fields including nuclear medicine. Several researchers described the potential use of nuclear medicine techniques for evaluating patients with COVID-19 (19). Furthermore, nuclear medicine will likely become a more important part of future antiviral drug development and treatment (20). On the other hand, the COVID-19 pandemic had a negative impact on imaging, not only for patient care but also for clinical trials (21).

Despite the well-recognized impact of the COVID-19 pandemic in clinical and research activities in nuclear medicine, researchers have adopted the mantra of “the show must go on.” To maintain the continuity of essential nuclear medicine services is considered a priority at the international level (2). Nuclear medicine education has been adapted to the pandemic setting (2). Furthermore, it is important to underline that the research activities in nuclear medicine will not end due to the COVID pandemic. Conversely, research activities are significantly growing considering, among others, innovating technologies, novel radiopharmaceuticals, emerging indications, and new methods of imaging/data analyses which will guarantee a bright future for the discipline.

## Author Contributions

The author confirms being the sole contributor of this work and has approved it for publication.

## Conflict of Interest

The author declares that the research was conducted in the absence of any commercial or financial relationships that could be construed as a potential conflict of interest.

## Publisher's Note

All claims expressed in this article are solely those of the authors and do not necessarily represent those of their affiliated organizations, or those of the publisher, the editors and the reviewers. Any product that may be evaluated in this article, or claim that may be made by its manufacturer, is not guaranteed or endorsed by the publisher.
